# Multiple limb compartment syndrome as a manifestation of capillary leak syndrome secondary to metformin and dipeptidyl peptidase IV inhibitor overdose: A case report

**DOI:** 10.1097/MD.0000000000021202

**Published:** 2020-07-17

**Authors:** Daisuke Kasugai, Kosuke Tajima, Naruhiro Jingushi, Norimichi Uenishi, Akihiko Hirakawa

**Affiliations:** a Department of Disaster and Traumatology; b Department of Emergency Medicine; c Department of Emergency and General Internal Medicine, Fujita Health University, Toyoake; d Department of Emergency and Critical Care Medicine, Nagoya University Graduate School of Medicine, Nagoya, Aichi, Japan.

**Keywords:** capillary leak syndrome, compartment syndrome, DPP-4 inhibitor, metformin

## Abstract

**Rationale:**

Capillary leak syndrome is a condition that increases systemic capillary permeability and causes characteristic manifestations such as recurrent hypovolemia, systemic edema, and hemoconcentration. Acute limb compartment syndrome is a possible complication of severe capillary leak syndrome. However, timely diagnosis and prompt treatment are challenging because of atypical presentation.

**Patient concerns:**

An 18-year-old woman with a history of clinical depression was admitted to our intensive care unit (ICU) because of metformin and vildagliptin overdose. She developed marked vasodilatory shock with recurrent severe hypovolemia and disseminated intravascular coagulation. After urgent hemodialysis and plasma exchange, she started to stabilize hemodynamically. However, her limbs became stone-hard with massive edema. Her serum creatinine kinase level increased to an extremely high level.

**Diagnosis:**

Extremities were distended, and her skin developed pallor with blistering. Intramuscular pressure in both forearms and lower legs was significantly elevated.

**Interventions:**

Decompressive fasciotomy was performed. Hemodialysis was continued because of rhabdomyolyses-induced acute kidney injury.

**Outcomes:**

The patient was finally able to walk by herself at the time of hospital discharge on day 109.

**Lessons:**

The possibility of acute compartment syndrome should be considered in patients with marked capillary leakage, especially after aggressive fluid resuscitation. It is important to be aware of the compartment syndrome in an ICU setting because communication barriers often mask typical symptoms and make diagnosis difficult.

## 1 Introduction

Capillary leak syndrome (CLS) is a group of diseases characterized by manifestations that are associated with systemic increase of capillary permeability.^[1]^ The typical presentation of CLS is hypotension/ hypovolemic shock, hemoconcentration, hypoalbuminemia, systemic edema, exudative serous cavity effusions, and noncardiac pulmonary edema. Hypercytokinemia is considered to result in an increase of capillary permeability, and disease severity differs based on the underlying cause.^[1]^ Although sepsis commonly shares these features, many other diseases can develop characteristics of CLS; drugs,^[2]^ hematopoietic stem cell transplantation,^[3]^ and viral hemorrhagic fever^[4]^ can all be triggers of CLS. Although severe and clinically significant forms of CLS tend to be accumulated in the intensive care unit (ICU) setting,^[1]^ the epidemiology of CLS remains undiscovered. From this perspective, CLS has not gained much attention so far. However, realizing this syndrome is important because some of the severe forms of CLS can develop complications that need prompt and appropriate treatment. Acute limb compartment syndrome is a good example that is accompanied by CLS and is a surgical emergency.^[5,6]^ Moreover, acute compartment syndrome accompanied by CLS shows atypical presentation, which make diagnosis challenging. Here, we report a case of severe CLS following a metformin and dipeptidyl peptidase-4 (DPP-4) inhibitors overdose, which resulted in multiple limb compartment syndrome.

## 2 Case report

An 18-year-old woman was transferred to the emergency department of our hospital because of loss of consciousness after an overdose of anti-hyperglycemic agents in a suicide attempt. She had been well until 11:00 pm 1 day before admission. Her grandmother found her unresponsive and lying on her belly at 6:00 am. Emergency medical service was called. She reportedly took her grandmother's pills: 64 tablets of metformin 250 mg (16,000 mg in total), 26 tablets of vildagliptin 50 mg (1,300 mg in total), 5 tablets of dapagliflozin-5 mg, 7 tablets of glimepiride-1 mg, 1 tablet of atorvastatin-10 mg, and some supplements (type and dose were unknown).

She had been attending a psychiatric clinic because of clinical depression and anorexia nervosa. She lived with her grandparents and was separated from her parents because she was sexually abused by her father. Approximately 5 months before admission, she tried to kill herself by jumping and was admitted to a psychiatric ward for 3 months. She reportedly did not intend to attempt suicide anymore at the time of discharge.

On arrival, she was unconscious and did not respond to pain stimuli. Her blood pressure was 94/56 mmHg and pulse was 70 beats/min. She had bradypnea with oxygen saturation at 100% while being on supplemental oxygen at 10 L/min via a reservoir mask. There was no rash or bruising on her skin. The remainder of the examination was normal. Her arterial blood gas showed lactic acidosis (pH 7.164, bicarbonate at 14.2 mmol/L, and lactate at 11.7 mmol/L) and hypoglycemia (glucose of 29 mg/dL). Blood creatinine level was elevated, and creatinine kinase level was normal. Other test results are shown in Table 1. The computed tomography performed on arrival was unremarkable except for slight infiltration of bilateral lower lung field, which is consistent with aspiration pneumonitis. The blood glucose level increased to 108 mg/dL after administration of 40 mL of 50% glucose. The trachea was intubated, and fluid resuscitation with 4 L of crystalloid was performed before starting norepinephrine. Supplementation with 40 mL of 8.4% bicarbonate was performed. Activated charcoal was administered via a nasogastric tube. Urgent hemodialysis was considered, and she was admitted to the ICU.

**Table 1:**
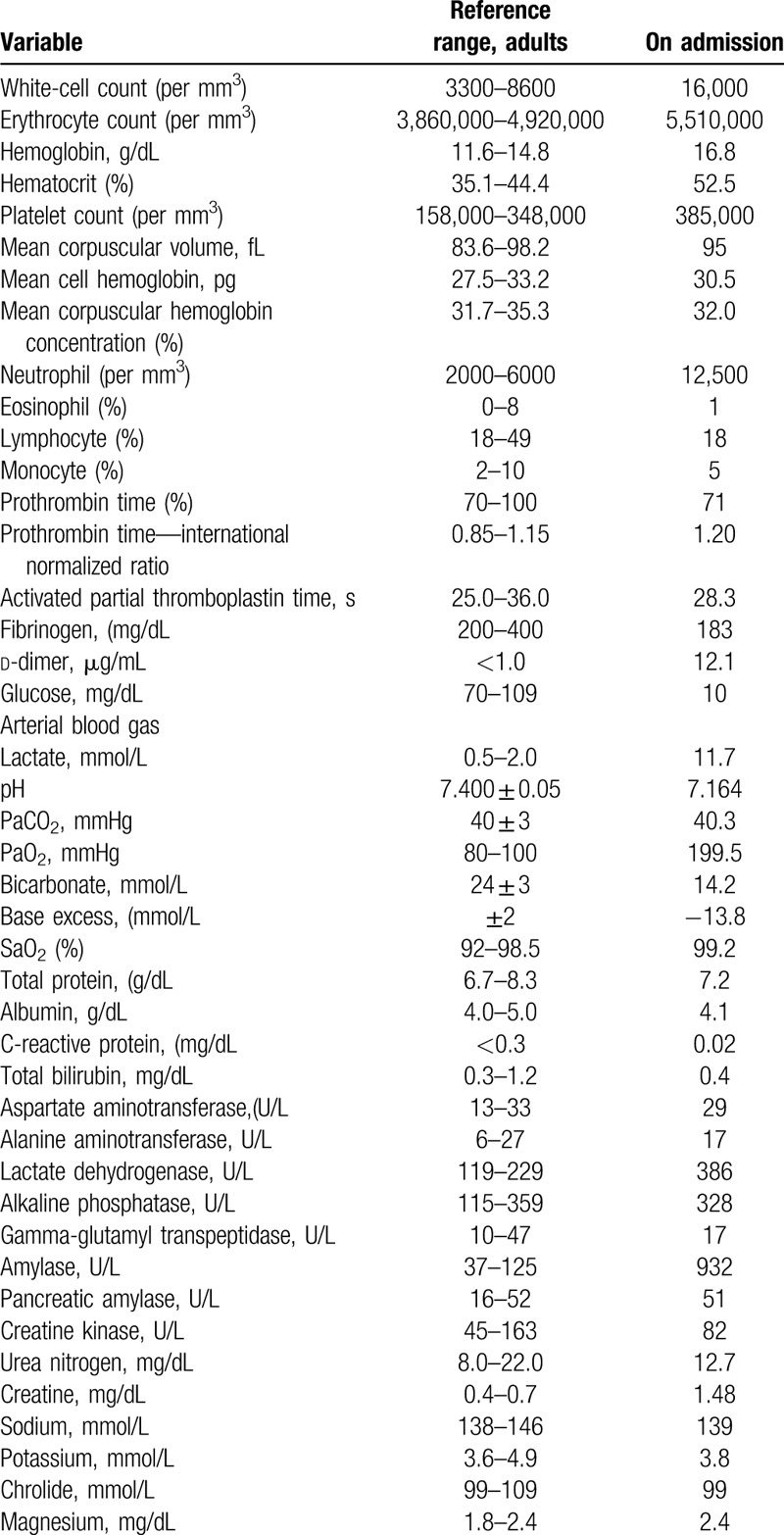
The details of laboratory findings.

**Figure 1:**
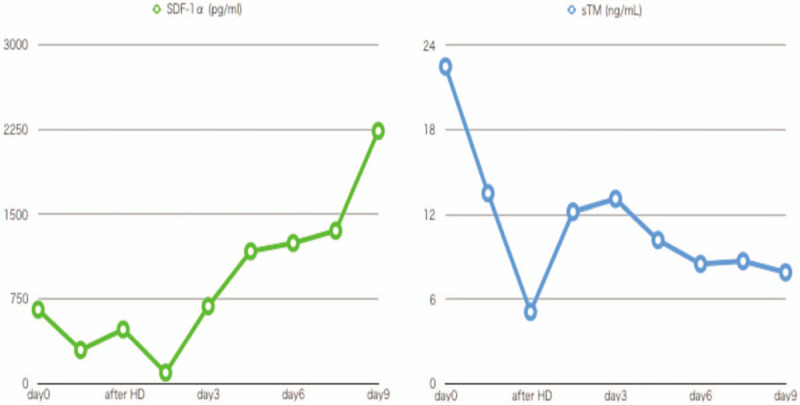
The trend of serum SDF-1α and sTM. The minimum of SDF-1α was 94 pg/mL on day 2, and the maximum of sTM was 22.5 ng/mL on admission. SDF-1α = stromal cell-derived factor-1α, sTM = soluble thrombomodulin.

After ICU admission, she developed recurrent hypotension despite aggressive fluid infusion and vasopressor support. Her blood pressure was becoming unstable with crystalloid infusion at 500 mL/h and stable with bolus infusion and albumin supplementation. For empirical antibiotics coverage, meropenem plus vancomycin was selected for possible septic shock. Hydrocortisone 200 mg was administered for possible relative adrenal insufficiency. The hemodynamic parameters obtained from Flotrac/Vigileo system (Edwards Lifesciences, Irvine, CA) immediately before hemodialysis were as follows: cardiac output (CO) 2.1 (reference range; 4.0–8.0) L/min, cardiac index (CI) 2.7 (reference range; 2.5–4.0) L/min/m^2^, and stroke volume variation (SVV) 37% (reference range; 10%–15%) with the use of norepinephrine at 0.7 μg/kg of body weight/min and vasopressin at 0.02 U/min. The bedside ultrasound showed collapsed inferior vena cava. She received sustained low-efficiency dialysis (SLED) using a polysulfone membrane, with blood flow rate of 150 mL/min, and dialysate flow rate of 300 mL/min. Three hours after initiation of hemodialysis, she developed overt disseminated intravascular coagulation (DIC) with platelet count of 27,000 cells/mm^3^, fibrinogen <35 mg/dL, prothrombin activity <10%, and d-dimer 31.3 μg/mL. Plasma exchange using 28 U of fresh frozen plasma frozen plasma was performed in tandem with SLED. After plasma exchange was completed, her hemodynamic status significantly improved: blood pressure 160/100 mmHg, pulse 120 beats/min, CO 9.0 L/min, CI 6.4 L/min/m^2^, and SVV 6% without using vasopressors. The antibiotics were discontinued after culture tests of the initial day were found to be all negative. The trends of plasma stromal cell-derived factor-1α (SDF-1α) as a marker of DPP-4 inhibitor's activity and soluble thrombomodulin (sTM) as a marker of endothelial injury measured after follow-up are shown in Figure 1.

**Figure 2:**
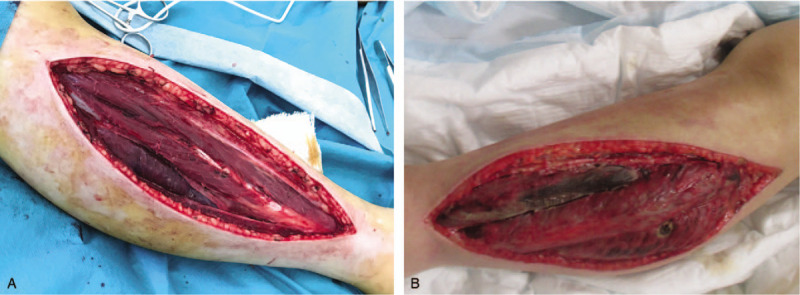
Patient legs after decompressive fasciotomy. Muscles of right (A) and left (B) legs were swollen and edematous.

**Figure 3:**
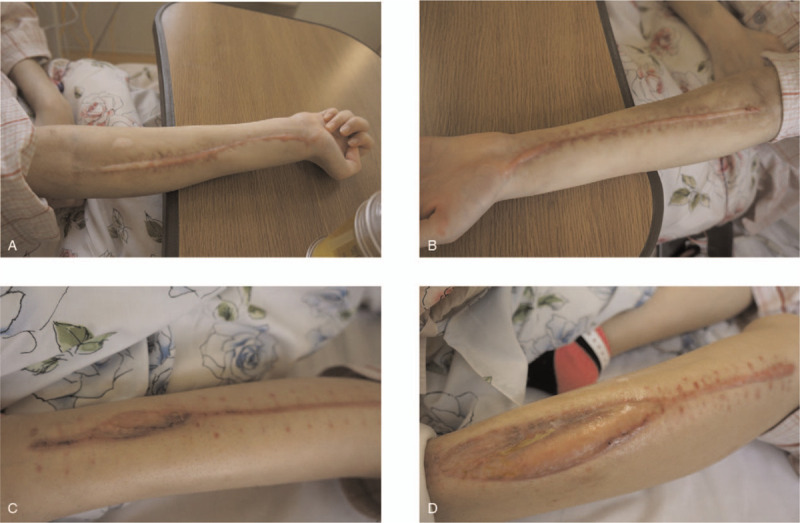
The extremities at the time of hospital transfer. (A) Right forearm; (B) left forearm; (C) right lower leg; (D) left lower leg. Both legs needed autologous skin transplantation.

On the following day, her creatinine kinase level increased to 41,560 U/L. Drug-induced rhabdomyolyses was suspected initially. However, her creatinine kinase increased up to 184,200 U/L on day 4 of admission. Her legs became stone-hard and were distended with massive edema. She also developed blisters on the arms. The details of intramuscular pressure of each limb are shown in Table 2. A diagnosis of acute limb compartment syndrome was made. Fluid overload was initially suspected as the cause of increased compartment pressure. Upper limb was observed to have fluid restriction and diuresis initially, and urgent decompressive fasciotomy of lower legs was performed (Fig. 2). However, the next day, compartment pressure of right and left upper limb rose up to 79 and 61 mmHg, respectively (Table 2). Decompressive fasciotomy of forearms was performed. The negative pressure wound therapy was introduced for management of massive edema and effusion. The wound was closed on day 31 with autologous skin transplantation. The operative wounds were cured at the time of hospital discharge (Fig. 3).

**Table 2:**
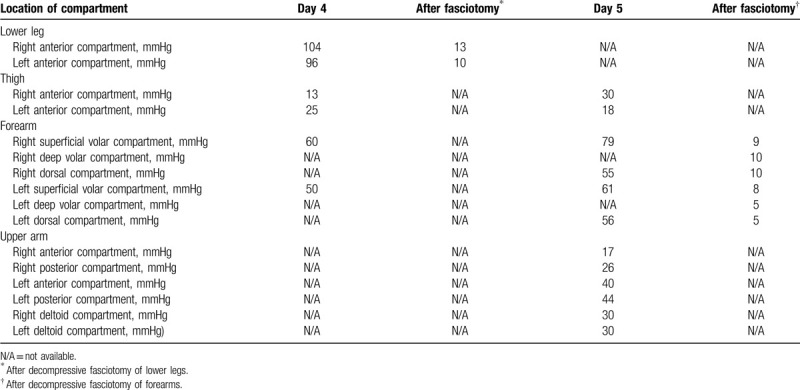
The result of intramuscular pressure.

**Figure 4:**
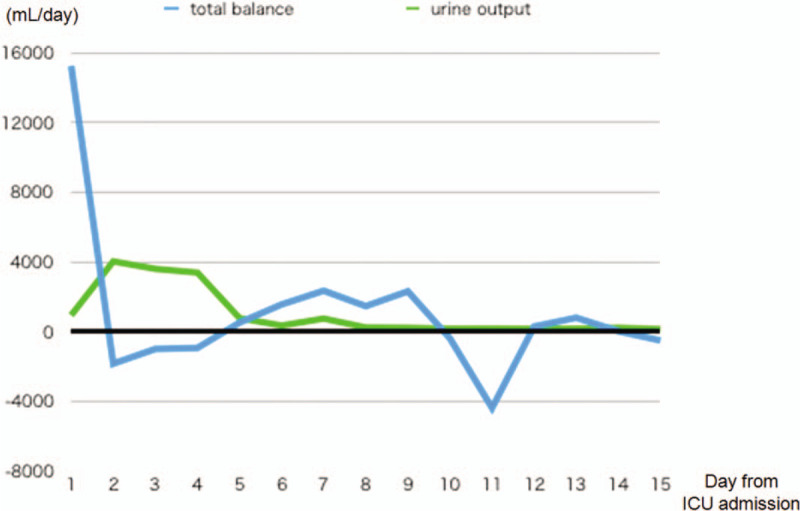
The trend of fluid balance after ICU admission. Net fluid balance turned negative from day 2 to day 4. The patient developed oliguria from day 6.

Following introduction of urgent hemodialysis, the patient developed rhabdomyolysis-induced acute kidney injury (AKI) when she was away from hemodialysis transiently. She was getting oliguric, and therefore, hemodialysis was restarted on day 10. The trend of fluid balance after ICU admission is shown in Figure 4. The patient was extubated on day 21. Intermittent hemodialysis was discontinued on day 34. She was discharged from the ICU on day 37. She complained of muscle weakness and could barely move to the wheelchair without assistance at the time of ICU discharge, but her muscle strength improved with rehabilitation. She was finally able to walk by herself at the time of hospital discharge, but bilateral paralysis of common perineal nerves and Volkmann ischemic contractures remained. No recurrence of hypotension nor compartment syndrome occurred after ICU discharge. She was transferred to another hospital for further rehabilitation and mental care on day 109.

## 3 Discussion

To the best of our knowledge, this is the first report of secondary CLS which is caused by metformin and DPP-4 inhibitor overdose. Also, this is the first report of compartment syndrome of all 4 limbs that resulted from secondary CLS.

### 3.1 Diagnosis and cause of CLS

The central pathophysiology of CLS is increased systemic vascular endothelial permeability and thereby protein leakage into interstitial space.^[1]^ Although there are no criterion standard diagnostic criteria of CLS, this patient showed severe recurrent hypovolemia and ongoing massive edema, hemoconcentration, AKI, and hypoalbuminemia that are consistent with CLS.^[7]^ The continuous fluid dependency and lack of other cause of hypovolemia (ie, bleeding, diarrhea and polyurea) are the main rationales for diagnosis of CLS in this patient. Increased SVV and collapsed inferior vena cava on echocardiogram are the suggestive findings of ongoing hypovolemia after aggressive fluid resuscitation. Although the development of DIC was not frequently reported in CLS cases, DIC in this patient was associated with systemic endothelial injury that was accompanied with CLS.^[8]^ The level of sTM, as a marker of endothelial injury,^[9]^ was extremely high on admission. Considering that sTM is associated with severity of DIC,^[10,11]^ the rapid development of DIC in this patient appears to be consistent with research findings.

The cause of systemic increase of capillary permeability was mainly due to DPP-4 inhibitor overdose. DPP-4 inhibitors are known to increase vascular leakage by augmenting the SDF-1α/VE-cadherin signaling pathway^[12]^ and cause angioedema.^[13]^ In this case, SDF-1α appears to be correlated with the severity of systemic capillary permeability. Interestingly, plasma SDF-1α decreased with the effect of vildagliptin, which seems to be a converse effect of DPP-4 inhibitors on SDF-1α. Although the reason is unclear, this finding is consistent with a previous report^[14]^ and may occur only with vildagliptin. The minimum level of plasma SDF-1α is on day 2, which is consistent with the pharmacokinetics of vildagliptin that the estimated volume of distribution is high (71 L) and that the fraction bound to proteins is relatively low (9.3%).^[15]^ The increase of sTM after plasma exchange also suggests that endothelial injury was sustained because the cause was not completely removed by hemodialysis and plasma exchange.

Other possible triggers of CLS include idiopathic, infection, ischemia-reperfusion injury, and metformin. Idiopathic CLS, as known as Clarkson syndrome,^[6,16]^ is a rare syndrome with less than 500 cases being reported so far. The typical presentation of Clarkson syndrome is recurrent episodes of increased capillary permeability with viral-like prodrome in the context of monoclonal gammopathy. Absence of typical presentation and no recurrence of episodes make the diagnosis of Clarkson syndrome unlikely in this patient. Severe infection may also result in sepsis, a well-known subtype of CLS.^[17]^ However, pneumonia in this patient was not severe enough to develop CLS. Ischemia-reperfusion injury as a result of sustained shock should be considered as a trigger of CLS.^[18]^ In this case, lactic acidosis caused by metformin overdose was partly associated with vasodilatory shock.^[19–21]^ However, systemic vascular leakage developed and continued from the initial phase of the shock, suggesting that shock is not the trigger of CLS. Besides, the dose of metformin was not high as in previous reports,^[19–21]^ indicating that metformin was not the leading cause of this extreme presentation. Interestingly, hemodynamic instability improved, not during SLED, but immediately after plasma exchange. This finding indicates that serum cytokine reduction, in addition to small molecule clearance, mainly contributed to hemodynamically improvement,^[22]^ which also supports the notion that metformin was not the main cause of the shock.

### 3.2 Diagnosis and cause of compartment syndrome

This patient developed compartment syndrome of all 4 limbs, which is a highly uncommon presentation of compartment syndrome other than CLS, and only idiopathic CLS^[23,24]^ cases were reported so far. The symmetrical change of intramuscular pressure also suggests that the trigger was a systemic effect rather than compression and atraumatic crush syndrome. To our knowledge, this is the first report of 4 limb compartment syndrome triggered by secondary CLS. Although idiopathic CLS is extremely rare, secondary CLS is commonly seen in ICU settings (ie, sepsis, post cardiac arrest syndrome). The compartment syndrome in ICU may be more common than we thought.

Loss of consciousness is a common presentation of overdose patients. The main signs of compartment syndrome (ie, pain, paresthesia, and paralysis)^[25]^ are thereby masked in this population, especially when they are intubated. Also, edema of the extremities is a common presentation of patients with fluid resuscitation. These factors make timely diagnosis and immediate decompression difficult. Rhabdomyolysis is often caused by medications,^[26]^ and creatinine kinase increase is a typical finding of ICU patients as a sign of rhabdomyolysis.^[27]^ However, the creatine kinase increase in this patient was extraordinal and the examination finding of tense compartments finally led to the diagnosis of compartment syndrome.

## 4 Conclusion

We report a case of secondary CLS after metformin and vildagliptin overdose and was complicated with 4-limb compartment syndrome. In patients with aggressive fluid resuscitation and creatine kinase increase, compartment syndrome should be included in differential diagnosis, and careful monitoring of compartment pressure may be needed.

## Acknowledgments

The authors thank all the ICU staff and nurses of the Disaster Trauma Center for treatment support, patient care, and rehabilitation.

## Author contributions

**Conceptualization:** Daisuke Kasugai.

**Analysis and interpuretation of data:** Daisuke Kasugai, Kosuke Tajima, Akihiko hirakawa.

**Writing – original draft:** Daisuke Kasugai.

**Writing – Review & Editing:** Kosuke Tajima, Naruhiro Jingushi, Norimichi Uenishi, Akihiko Hirakawa.
